# The Effect of Slaughter Age on Meat Quality of Male Kids of the Polish Carpathian Native Goat Breed

**DOI:** 10.3390/ani12060702

**Published:** 2022-03-11

**Authors:** Aldona Kawęcka, Marta Pasternak

**Affiliations:** Department of Sheep and Goat Breeding, National Research Institute of Animal Production, 32-083 Balice n. Cracow, Poland; marta.pasternak@iz.edu.pl

**Keywords:** Carpathian goat, native breed, meat quality, slaughter age, kids

## Abstract

**Simple Summary:**

The study aimed to compare the carcass measurements and the quality and composition of meat from male kids of the Polish Carpathian native goat breed slaughtered at the ages of 9 and 12 months. A dressing percentage was higher in older kids than in younger ones, as well as final weight and cold carcass weight. The weight of valuable cuts differed between groups, and it was significantly higher in 1-year-old kids. No differences were found in basic ingredients, such as moisture, protein, ash and vitamins A and E, depending on the kids’ slaughter age. Significant differences occurred in relation to the physicochemical parameters, fatty acid profile and organoleptic properties. Roasted meat from the Carpathian kids scored high marks in the organoleptic assessment and the meat obtained from older animals was rated higher.

**Abstract:**

The native breed of Carpathian goats, once abundant in the foothills of Poland, practically died out and was replaced by other, more efficient breeds. As a result of reintroduction activities and its inclusion in the genetic resources program, breeding was restored. The dynamically developing population of Carpathian goats is an extremely valuable element of biodiversity and a potential for the development of the market for its products, including goat meat. The study aimed to compare the carcass measurements and the quality and composition of meat from male kids of the Polish Carpathian native goat breed slaughtered at the ages of 9 and 12 months. Muscle samples were taken from the leg (m. biceps femoris) to determine the meat chemical composition and physicochemical and sensory parameters, as well as the fatty acid profile. The dressing percentage was higher in older kids (41.27%) than in younger ones (37.89%), as well as final weight and cold carcass weight. The weight of valuable cuts such as the loin and leg differed between groups, and it was significantly higher in 1-year-old kids. No differences were found in basic ingredients, such as moisture, protein, ash and vitamins A and E, depending on the kids’ slaughter age. The fat concentration was significantly higher in the group of younger kids. Significant differences occurred in relation to the physicochemical parameters, fatty acid profile and organoleptic properties. The findings demonstrated that the meat of older kids was characterised by darker colour and a slightly higher pH, and it contained a higher concentration of hypocholesterolemic fatty acids (DFA) and a more favourable DFA/OFA ratio. Roasted meat from the Carpathian kids scored high marks in the organoleptic assessment and the meat obtained from older animals was rated higher.

## 1. Introduction

The goat population in Poland is small, and the vast majority includes dairy breeds. Goat milk products enjoy considerable popularity and are now a firm fixture in the diet of a number of consumers, and their range is very wide—from drinking milk, rennet and cottage cheese, fermented drinks, cream, and condensed and powdered milk to rice porridges, butter and even sweets. Agritourism and ecological farms have their own, original dairy products on offer, as well as increasingly more goat products, obtained from young male animals [1]. Goat meat in Poland is rarely available, and the demand for it is limited to consumers in larger cities and is associated with a small group of gourmets and restaurants that serve mainly Middle Eastern foods. Goat meat, like meat from other species, may be classified according to the age or weight at slaughter, and the name may depend on the geographical area. The general appellation is “goat”, although adult goat meat is referred to as “chevon”, and young goat meat as “kid”. The names “capretto” from Italian and “cabrito” from Spanish and Portuguese are also known. “Capretto” stands for meat from carcasses having a weight of 5–12 kg obtained from 4–7-week-old suckling kids [2,3]. In the 1920s, the United States Department of Agriculture (USDA) promoted the term “chevon”, or “portmanteau of chèvre” (“goat”) and mouton (“sheep”, “mutton”), which stands for carcasses having a weight of about 16–22 kg obtained from older animals, about 3–9 months old [4].

Until recently, the Boer goat was the most frequently represented meat breed covered by the utility assessment in Poland [5]. In 2015, their percentage in the active population in Poland was 34.5%, but in the same year, native Carpathian goats began to gain popularity. In 2018, they actually accounted for almost half of the assessed part of the population [5]. Currently, Carpathian goats can be found most often in southern Poland. This native breed, once abundant in the foothills of Poland, has practically become extinct and been displaced by other, more efficient breeds. Intensive reintroduction activities towards the Carpathian goat and its inclusion in the genetic resources programme [1] have triggered an increase in its population. As a breed of versatile utility (milk, meat, skins), the role of the Carpathian goat has become increasingly prominent over recent years, which provides an opportunity to develop the market for products obtained from it, including goat meat.

The study aimed to compare the carcass measurements, physicochemical parameters, fatty acid profile, and organoleptic properties of the meat from male kids of the Polish Carpathian breed slaughtered at the ages of 9 and 12 months.

## 2. Materials and Methods

### 2.1. Animal Material, Diet and Slaughter Evaluation

The experiment was carried out on the farm of the National Research Institute of Animal Production (NRIAP). The research material consisted of Carpathian kids from a herd belonging to the NRIAP farm located in the southern part of Poland. All of the goats were from twins. Kids on the farm were kept with their mothers until about 60 days of age, where they received crushed oats and hay. After weaning at 90 days of age, animals were fed with good quality hay and received daily 200 g of a concentrated mixture containing: 52% barley, 20% oats, 5% wheat bran, 15% soybean meal, 5% rapeseed expeller, and 3% mineral mixture. Twenty Carpathian male kids at the age of 6 months were selected for the experiment and placed in the NRIAP livestock buildings. The kids were kept in one group; the area of the pens was 1.5 m^2^ per individual. After 10 days of adaptation, the fattening period started, which lasted from 7 months to slaughter at 9 and 12 months. During the fattening period, the kids were fed with hay ad libitum and received daily 400 g of the same concentrated mixture. The animals had constant access to fresh water and mineral licks. Slaughter was performed at an EU-licensed abattoir. Procedures met the requirements of the Directive 2010/63/EU of the European Parliament and of the Council of 22 September 2010 on the protection of animals used for scientific purposes. After 24 h of chilling at 4 °C, the evaluation of meat performance included post-slaughter carcass evaluation and determination of the proportion of carcass cuts performed according to the methodology for small ruminants prepared by the National Research Institute of Animal Production [6]. The carcasses were separated into cuts that were subjected to detailed dissection into meat, fat and bone tissues. Samples used in the experiment were taken from commercially slaughtered kids. Muscle samples were taken from the lateral face of the right hind limb (m. biceps femoris from the leg) to determine chemical composition, physicochemical and sensory parameters, and the fatty acid profile of the meat.

### 2.2. Meat Physicochemical Parameters

The following parameters were determined in a raw muscle, m. quadriceps femoris. pH45—pH value after 45 min from slaughter—was determined using a Hanna Instruments HI99163 pH meter equipped with an FC232D stylus electrode (Merck KGaA, Darmstadt, Germany). L*, a* and b* colour parameters 48 h after slaughter were measured using a Minolta CR 400 apparatus (Konica Minolta, Osaka, Japan), according to the CIE system, taking into account brightness L*, redness a* and yellowness b*. Drip loss (%) was determined relative to the initial sample weight (before packaging). The cooking loss (in %) was determined from the difference in weight of the sample before and after a 60 min thermal treatment in a water bath at 70 °C.

Meat was vacuum-packed and frozen at −21 °C until analysis. Chemical composition analyses were performed using standard methods, according to AOAC [7]. The following parameters were determined: moisture, total protein, ash and fat. Fat was extracted by means of Folch [8]. Vitamins A and E were quantified using HPLC (Merck-Hitachi, Tokyo, Japan) [9] with UV-Vis detection (324 nm) for vitamin A and fluorescence detection (Ex295 nm, Em350 nm) for vitamin E. The samples were saponified (70 °C, 60 min) in the presence of 60% KOH and ethanol, followed by extraction with ethyl acetate:hexane (1:9). After evaporation of the solvents under nitrogen (40 °C), the residue was dissolved in ethanol and determined by reversed-phase HPLC using a LiChroCART™ 250-4 Superspher^®^ 100 RP-18 (4 μ) column (Merck, Darmstadt, Germany) and solvents (methanol:water, 96.5:3.5, *v*/*v*) for elution (1 mL/min). Before analysis, calibrated external vitamin standards (Sigma, St. Louis, MO, USA) were used. The method determines the content of vitamin A in the form of all-trans-retinol and vitamin E in the form of alpha-tocopherol.

Fatty acid composition in intramuscular fat from kid’s leg meat was analysed with a VARIAN 3400 gas chromatograph (Varian, Walnut Creek Instrument Division, Walnut Creek, CA, USA) with an Rtx 2330 column (dimensions 105 m × 0.32 mm × 0.2 μ; column operating temperature: initial 60 °C for 10 min; temperature rise up to 120 °C (20 °C/min); up to 240 °C (3 °C/min), analysis time: 60 min, dispenser temperature: 250 °C; detector: 250 °C; carrier gas: helium, 3 mL/min.; injection of 1.0 mcl). Samples were extracted with the chloroform/methanol mixture (2:1, *v*/*v*). Fatty acids were saponified (0.5 N NaOH in methanol, 80 °C) followed by esterification with boron trifluoride/methanol. After extraction with hexane, the compounds were separated on the RTX-2330 column (105 m, 0.32 mm, 0.2 µ; flow rate 4 mL/min; injection 1 μL) using a flame ionisation detector and autosampler AOC-20i ((both Shimadzu, Kyoto, Japan). The temperature programme in the column oven was 60 °C to 120 °C (at a rate of 20 °C/min), and then 240 °C (at a rate of 3 °C/min). Fatty acids were identified by comparing their retention times to those of methylated FA standards (Supelco Inc., Bellefonte, PA, USA).

The maximum shear force was measured with a Warner–Bratzler V slot blade (shearing blade thickness of 1.016 mm; V-shaped cutting blade with a 60 degree angle) on a texture analyser Model TA-XT2 plus equipped with a 1 kN cutting head (Stable Micro Systems, Godalming, Surrey, UK). The meat samples were cut from the cooked muscles (the meat was prepared the same as for the determination of cooking losses) into 10 mm × 10 mm × 40 mm cubes (minimum of 3 per sample). Each sample was cut in half, parallel to the muscle fibre orientation on a Heavy Duty Platform (HDP/90) (2 mm/s test speed).

### 2.3. Sensory Evaluation

Samples of leg meat were thawed at 4 °C the day before the panel sensory session. The samples were wrapped individually in cooking bags and roasted in an oven until the internal muscle temperature reached between 70 and 80 °C. The roasted leg meat was organoleptically evaluated for aroma, taste, juiciness, and tenderness according to the method of Baryłko-Pikielna and Matuszewska [10]. A 5-point grading scale (1 = lowest to 5 = highest) was used: aroma: 1—very undesirable, 5—very desirable; juiciness: 1—very dry, 5—very juicy; tenderness: 1—very tough, 5—very tender; taste: 1—not tasty, 5—very tasty. For each parameter, the scores were accurate to 0.5 point. The sensory panel consisted of seven members trained in sensory profiling according to ISO 8586:1993.

### 2.4. Statistical Analysis

The results were statistically analysed with the use of STATISTICA package, v. 13.1, 1984–2016 Dell Indust. and one-way ANOVA (‘analysis of variance‘) in order to evaluate the effect of age at slaughter on carcass measurements and the quality and composition of kid’s meat [11]. All values are expressed as means and standard deviation. Multiple mean comparisons and the significance of the differences were estimated using Duncan’s test. Testing was carried out at the significance levels *p* ≤ 0.05 and *p* ≤ 0.01.

## 3. Results

The selected fattening and slaughter parameters of kids are presented in Table 1. The initial weight of kids was 15.58 kg; the weight at the end of the fattening period was 24.06 kg for kids at the age of 9 months and 30.71 kg for older animals (*p* < 0.05). Daily gains during the fattening period were 142.76 g for younger kids and 103.8 g for kids at the age of 1 year. The slaughter parameters such as cold carcass weight and dressing percentage differed significantly between the two groups. The dressing percentage was 37.89% for younger and 41.27% for older kids. Weight of valuable cuts differed between groups for the saddle and leg. The results of leg dissection showed differences (*p* < 0.01) in the bone weight; no significant differences between groups were found for meat and fat weight.

Table 2 illustrates the results focusing on the content of nutrients and vitamins A and E in the analysed meat.

The average moisture content in the tested goat meat was 76.54%, and the average content of protein and ash was 20.84% and 1.18%, respectively. Vitamin A content was 0.13–0.32 µg/g, while vitamin E ranged between 1.17–1.32 µg/g. Only the fat concentration differed significantly, and it was higher in the younger kids (2.27%) than in the older ones (1.95%).

Table 3 presents the physicochemical properties of kid’s meat. The meat of older animals had a significantly higher (*p* ≤ 0.01) pH value 45 min after slaughter, amounting to 6.54, while the pH of meat from younger animals was 6.27. Assessment of meat colour revealed statistically significant differences (*p* ≤ 0.01) in brightness (L*). The meat of the older kids was darker (42.22) than that of the younger kids (44.48). The yellowness (b*) was also lower in the meat of 12-month-old male kids (2.98) than in the meat of 9-month-old ones (3.84). There were no statistically significant differences between the groups in the redness (a*) value. The samples did not differ significantly in terms of drip loss, cooking loss and the shear force applied in the heat-treated Carpathian goat meat. Drip loss ranged from 0.10–0.12%, while cooking loss was 33.58% in the meat samples from the younger kids and 34.11% in the meat samples from the older kids. The shear force did not differ significantly between the groups and averaged 70.19 N.

Table 4 illustrates the fatty acid profile in the tested kid’s meat. The meat from both groups was characterized by a higher concentration of unsaturated fatty acids (>60%) than of saturated fatty acids. At the same time, no differences were found between the groups in terms of SFA and UFA content. The meat of the younger kids contained significantly more (*p* ≤ 0.05) MUFA acids, while the meat of the older animals contained more (*p* ≤ 0.01) polyunsaturated acids (PUFA), including the *n-6* PUFA fraction. As a result, the group of the older kids had a higher (*p* ≤ 0.01) PUFA/SFA ratio and a higher (*p* ≤ 0.01) PUFA*n-6*/PUFA *n-3* index. The meat of the 9-month-old kids was found to have a significantly higher (*p* ≤ 0.01) concentration of palmitic (C16), oleic (C18-1) and erucic (C22-1) acids than the meat of the 12-month-old kids, which in turn had a significantly higher (*p* ≤ 0.01) concentration of linoleic acid (C18-2), arachidic acid (C20), arachidonic acid (C20-4), and behenic acid (C22). The samples of meat from the younger group had a significantly higher concentration of CLA c9-t11 linoleic acid isomers, while the samples from the older kids had a higher (*p* ≤ 0.01) concentration of CLA c9-c11. The content of hypocholesterolemic fatty acids (DFA) was significantly higher (*p* ≤ 0.01) in the samples collected from the 12-month-old kids, whose meat also contained significantly less hypercholesterolemic fatty acids (OFA). This translated into a higher (*p* ≤ 0.01) DFA/OFA ratio in this group of kids than in the 9-month-old kids.

Table 5 illustrates the results of the organoleptic analysis performed on the tested meat samples. Statistically significant differences (*p* ≤ 0.01) were found with regard to all of the assessed traits: the aroma of the baked leg, juiciness, tenderness and taste. The qualities were rated high (over 4 points) in both groups. The evaluators assessed all meat features higher for the group of older animals. The meat of the 12-month-old kids was more aromatic, more tender, and juicier. The testers described the meat as “very tasty”.

## 4. Discussion

The world goat population has grown significantly in recent years. In 2006–2016, the world population of goats increased by 19.3%, while that of cattle increased by 6.7%. The greatest rise was observed in Africa and Asia, where goats’ adaptability to harsh environmental conditions has made them a very popular species of livestock [12]. The global meat market is developing, and typical meat goat breeds (Boer), interracial crossbreeds, as well as goats of native breeds, for example, the Carpathian goat in Poland, are used for the production of goat meat. Consumers increasingly appreciate products that are not only tasty but also healthy, such as kid’s meat. Goat meat is low in fat and is also relatively high in protein and mineral salts. It contains an abundance of iron, potassium and fatty acids, which play a major role in the proper functioning of the human body [13,14]. Goat meat has a nutritional value similar to that of sheep meat [15], but its fine-fibre structure makes it easier to digest. Due to the low content of saturated fatty acids and cholesterol and a high concentration of polyunsaturated fatty acids, as well as the presence of valuable amino acids such as lysine, threonine and tryptophan, goat meat can be a healthier alternative to other red meat varieties [16,17].

Dressing percentage, which is one of the most important characteristics determining the small ruminant’s slaughter value, depends on numerous factors, such as age, weight at slaughter, housing system, breed and sex. In the presented research, the dressing percentage achieved by kids was 37.89%, by younger 41.27%, and it differed between the two groups (*p* ≤ 0.05). According to Webb [18], the dressing percentage of goat carcasses tends to be less than that of sheep, mainly due to the reduced carcass fat content of goats, and average dressing percentage varies between 50 and 55%. In suckling kids, it can reach higher values of up to 61.5% [19]. According to Marichal et al. [20], dressing percentage was in the range of 40–47%, depending on the age and weight of the kids; higher values were found for younger kids. In Rajkumar et al. [21], age had no significant effect on dressing percentage in 9- and 12-month-old Sirohi kids under semi-intensive and intensive systems of management (45.77–47.61%), whereas at 12 months, the kids maintained under an intensive system of management had a significantly higher dressing percentage (48.13 vs. 45.53%). The results obtained in the presented study were similar to those of Kiani and Fallah [22] for Iranian native Lori goats whose slaughter weight was 20 kg and 30 kg (39.3 vs. 42.7%). Daily gains of Carpathian kids did not differ significantly depending on the age at slaughter (103.88–142.76 g). For Boer goats, which are a meat breed, the daily gains of 2-month-old kids were about 200 g [5].

The use of cuts of a goat carcass varies from country to country; in western countries, cuts from the lumbar region, dorsal part of the chest, and hind limbs are most sought after. In contrast, some African and Asian studies have shown a high preference for cuts from the breast area [18]. In the case of Carpathian kids, higher weight of steak and loin was found in older kids. Pieniak-Lendzion et al. [23] found such relationships for all valuable cuts. Dissection results for the leg showed that the bone tissue content in older goat kids was more than twice as high as that in younger kids. Pieniak-Lendzion et al. [23] revealed that the bone tissue content was lower in older goat kids, whilst the fat tissue content was higher.

The chemical composition of goat meat is influenced by breed, sex, age, nutrition and health status, as well as the level of stress and the slaughter procedure itself [24]. The quality of Carpathian goat meat in comparison with that of the Saanen breed was presented by Migdał et al. [25], who found differences between breeds: meat from the Carpathian goats was characterised by a lower shear force and hardness; therefore, it was more juicy and tender compared to meat from the Saanen goats. Furthermore, the Carpathian goat meat contained more essential amino acids and its fat was characterised by a higher content of monounsaturated acids and a more favourable (lower) saturation index.

In the presented study, the slaughter age of the kids had no effect on the chemical composition of the meat, such as moisture, protein or ash. Only the fat concentration was significantly higher in the 9-month-old kids than in the 12-month-old ones. Differences in dry matter, crude protein and fat were found by Mehjabin et al. [26] in the meat from Black Bengal goats of both sexes; however, these studies involved a greater age difference between animals—1-year-, 2-year- and 3-year-olds were compared. The examined samples differed significantly in the content of dry matter and fat, which increased with the age of the slaughtered animals, and the level of protein, which decreased with age. The analysis of chemical composition in the examined kid goat meat showed a low fat content in both age groups. The tested samples contained on average 2.11% fat, which is a concentration similar to the results obtained by Arain et al. [27], of 2.52%. The fat content in the meat of the 9-month-old Carpathian kids was similar to that found in goats aged 8–10 months in the study conducted by the above-mentioned authors (2.71%). In the samples from the 12-month-old Carpathian kids, the fat content was determined at a slightly lower level, proving that meat from older animals is as valuable as that from younger ones. The moisture and ash content, averaging 76.54% and 1.18%, respectively, were similar to the results obtained by other authors [13,23,27]. Likewise, the average protein concentration of 20.84% was similar to the results obtained by Ivanovic et al. [13] for the Balkan goat and the Serbian White goat—20.25%. Marichal et al. [20] analysed the quality parameters of meat collected from various muscles of Canary Caprine goats weighing 6, 10 and 25 kg, and found no differences in the content of basic ingredients. The moisture content was in the range of 76.30–78.55%, protein 18.55–20.75%, fat 0.96–1.3%, and ash 1.08–1.16%.

Goat meat is characterised by a high concentration of protein and minerals and low content of fat [17,28]. It is also a source of vitamins, especially vitamins of the B group (B3, B6, B9, B12) as well as vitamins A, E, C and K [29]. The average concentrations of vitamin A and E in the examined meat samples were 0.23 and 1.25 µg/g, respectively. Del Mar Campo et al. [29] also analysed the concentration of vitamins in the meat of suckling kids and lambs. The content of vitamin A was too low to be detected, and the content of vitamin E (α-tocopherol) in suckling kids was 2.3  µg/g, which was 2 times more than in the presented study. This difference may be due to the goat breed, but it can also result from the large age difference between the studied kids who were 9 and 12 months old in our research and only 1 month old in the research of the aforementioned authors.

The physicochemical properties of kid’s meat can be influenced by various factors. Differences between production systems were found to be significant for meat pH, water holding capacity, cooking loss, tenderness and even some colour characteristics [30]. In the presented results, statistically significant differences (*p* ≤ 0.01) were noted in relation to the meat pH, which was characterised by a higher value in older goats (6.54), as in Toplu et al. [31], who obtained pH values increasing with the age and weight of goats slaughtered at 3, 6, 9, and 12 months (6.37–6.53). Similar results of significantly higher pH in older kids were obtained by Rajkumar et al. [21], who compared the meat parameters in 9- and 12-month-old goats, depending on the intensity of feeding. Pieniak-Lendzion et al. [23] obtained a pH value of 6 for younger goats and 6.03 for older ones. The pH value testifies to the quality of the meat, which translates into its culinary utility, and the mean pH (6.4) obtained in these studies fell within the range adopted for meat immediately after slaughter and corresponded to the values obtained by Stanisz et al. [32] in studies conducted on Boer goats and crossbreeds (pH45—6.35–6.45). Another feature that differed between the groups was the lightness of colour L*, which turned out to be significantly higher (*p* ≤ 0.01) in younger animals than in older ones, whose meat was darker. Some authors point to a relationship between the pH value and the L* parameter, where the increasing level of pH reduces the brightness (L*) and vice versa [33]. This relationship was reflected in our own results, where a significantly lower pH in the meat of younger kids was associated with a significantly higher L*, a lighter colour of the meat, and with a significantly higher value for the yellowness (*p* ≤ 0.01). When making purchasing decisions concerning meat, consumers are often guided by the colour according to which it is classified, but also use it to judge the product freshness [34]. There were no significant differences in the shear force, averaging 70.19 N, which was higher than that in the younger kids tested by Borgogno et al. [3]. Differences in shear force depending on the weight of the kids (age at slaughter), but also on the type of muscle, were found by Marichal et al. [20], who analysed the parameters of Canary Caprine kid goats weighing 6, 10, and 25 kg. The highest shear force of 68.42 N, compared to that of the group of younger animals (mean 50 N), was recorded in the semimembranosus muscle from the group of the oldest goats. For the longissimus muscles, the shear force was 80.99 N compared to 56.4 N in the younger goats, while the authors found high N values (90.15 N) in all age groups for the triceps brachii.

The type and composition of fatty acids determine the dietary value of meat. Polyunsaturated fatty acids play a particularly considerable role, as they counteract and alleviate the course of a number of conditions, commonly referred to as civilisation diseases, such as coronary artery disease, autoimmune diseases and cancer [35]. The intramuscular fat of the leg muscle in both groups had a higher concentration of unsaturated fatty acids (over 60%) than that of saturated fatty acids, similar to the results of Pieniak-Lendzion et al. [23], in which the UFA profile was 60.78% in 3-month-old male kids and 58.4% in 6-month-old kids. There were no differences between the groups in terms of SFA and UFA values. Similar results were obtained by Sikora and Kawęcka [36] for Carpathian and white ennobled goats. The meat of the younger goats contained significantly more (*p* ≤ 0.05) MUFA acids, while the meat of the older goats contained more polyunsaturated acids (PUFA), including *n-6* PUFA. These values were reflected in significantly higher (*p* ≤ 0.01) ratios of PUFA/SFA and PUFA 6/3 in the group of older goats, amounting to 0.86 and 11.26, respectively. Banskalieva et al. [37] used the collected literature data to provide a lower range of values for PUFA/SFA (0.16–0.49) depending on the examined tissue, averaging 0.33. The age-dependent change in the concentration of polyunsaturated fatty acids was confirmed by the results of Dhanda et al. [38], who found a significantly higher PUFA content in older goats (30–35 kg), intended for the production of chevon carcass, than in younger goats (14–22 kg), intended for capretto carcass. Our own research also revealed a significantly higher (*p* ≤ 0.01) content of CLA c9-t11 isomer in the meat of younger kids. A similar relationship for this isomer was found by Sikora and Kawęcka [36], who determined the value of CLA in the meat of 120- and 180-day-old Carpathian kids, amounting to 1.06 and 0.47 mg/100 g, respectively. Conjugated dienes of linoleic acid are valuable for the human body, as they stimulate the immune system and have antioxidant and anti-atherosclerotic properties [39]. The main isomer C18:2 cis-9 trans-11 exhibits anti-cancer properties and has a positive effect on diabetics; therefore, many studies have been conducted to increase the concentration of CLA in ruminant products [40]. Significant differences (*p* ≤ 0.01) were observed in the content of oleic (C18-1) and linoleic acid (C18-2), i.e., the meat of younger kids was characterised by higher concentration of C18-1, while the meat of older ones contained more C18-2. Toplu et al. [31] wrote about similar differences between 9- and 12-month-old kids, as they obtained slightly lower values for these acids. In the analysed samples from the Carpathian kids, C18-1 acid constituted 40% of all determined fatty acids, which is reflected in the results of Pieniak-Lendzion et al. [23] and in the results of the authors cited in Banskalieva et al. [37], who compared the fatty acid profiles in the adipose tissue of goats and other ruminants. The share of palmitic acid in the analysed goat meat was significantly higher (*p* ≤ 0.01) in the group of younger animals (18.52 g/100 g) than in the older group (17.10 g/100 g), which could also be found in the results presented by the aforementioned authors, who determined this acid at a higher concentration in 3-month-old than in 1-year-old kids [31]. The meat of younger goats contained a significantly lower concentration (10.65 g/100 g) of arachidonic acid (C20-4) than the meat of older goats (14.13 g/100 g), unlike Mahgoub et al. [41], who described decreasing content of arachidonic acid in the muscles of 11 kg (1.61%), 18 kg (0.65%) and 28 kg (0.09%) goats.

DFA content in the tested meat samples of Carpathian kids was significantly higher (*p* ≤ 0.01) in 1 -year-old kids than in 9-month-old ones, while OFA was significantly lower (*p* ≤ 0.01). Theses DFA and OFA values translated into the differences in the DFA/OFA ratio, which were significantly higher (*p* ≤ 0.01) in the older (5.2) than in the younger (3.9) Carpathian kids. A high ratio of DFA/OFA proves that the examined goat meat can be considered a product of high nutritional value [14]. Therefore, it can be concluded that the meat of older goats was characterised by a more favourable content of hypocholesterolemic fatty acids (DFA) and a more favourable DFA/OFA ratio than the meat of younger goats. It must be mentioned that the examined kid’s meat was characterised by a favourable UFA/SFA ratio of 1.78–1.88, higher than that in Dhanda et al. [38], where it was 0.47 for younger and 0.97 for older animals. Similar UFA/SFA ratios in 3- and 6-month-old Carpathian kids, amounting to 1.54 and 1.26, respectively, as well as 1.39 and 1.56 in Boer kids, were obtained by Sikora and Kawęcka [36]. In turn, in the studies by Ivanovic et al. [13,14], 4-year-old goats were found with a ratio of 0.92 and 0.96, and 0.81–0.85 depending on the breed (Balkan, Alpine, Saane). In the Omani breed [41], the UFA/SFA ratio increased with the age and weight of the kids, similarly to the kids slaughtered at the age of 3, 6, 9, and 12 months in the study by Toplu et al. [31], where these values increased in the range of 0.83–1.04. The different values indicate major differences in the fatty acid profile depending on breed and age at slaughter.

Organoleptic analysis of the meat from the examined leg, derived from Carpathian kids, showed statistically significant differences (*p* ≤ 0.01) depending on the age at slaughter in terms of all assessed parameters: aroma, juiciness, tenderness, and taste. All parameters were rated above 4 points, which is a high score according to the scale used in the analysis, as in the case of Pieniak-Lendzion et al. [23], who evaluated the meat from white ennobled kids. Contrary to prevailing opinions about the specific, unpleasant “goat” odour and taste, concerning particular meat derived from non-castrated males, the Carpathian kid’s meat tested in the present research was described as “very tasty”, whereby the meat from the older, 12-month-old kids was rated higher. In terms of taste, aroma and tenderness, the above-mentioned authors [23] also obtained significantly higher results for meat from older kids. Stanisz et al. [32] found significant differences in the tenderness and juiciness of goat meat depending on breed, with no differences in taste and smell, while Tshabalal et al. [42] showed major differences in smell and taste in meat samples from the Boer goat and South African native breeds.

## 5. Conclusions

The presented study demonstrated that slaughter age (9 and 12 months) had a significant effect on carcass measurements, physicochemical parameters, fatty acid profile, and organoleptic properties of meat from Carpathian kids. Older kids were characterised by higher dressing percentage (41.27%) than younger ones (37.89%), and they achieved higher final weight and cold carcass weight. The weight of valuable cuts such as loin and leg differed between groups, and it was significantly higher in 1-year-old kids. The fat concentration was significantly higher in the group of younger kids. The results imply that the meat of the group of older (12-month-old) kids was darker and had a slightly higher pH. In the samples from both age groups, we obtained a high share of UFA: 63.93% in the younger and 65.12% in the older kids, which serves as an important indicator for consumers looking for healthy food. Meat from the younger goats was characterised by high content of MUFA (40.72%) and CLA c9-t11 (0.58%), while meat from the older goats was rich in PUFA (29.74%). This points to the high nutritional value of meat from both less and more mature animals. Furthermore, the meat of the older kids contained a higher concentration of hypocholesterolemic fatty acids (DFA) and a more favourable DFA/OFA ratio. It should be emphasized that the meat of Carpathian kid goats, as a product derived from a native breed, obtained high organoleptic results, which is an argument to convince consumers of not only the exceptional pro-health properties but also the culinary value of goat meat.

## Figures and Tables

**Table 1:** Selected parameters of fattening and slaughter performance of kids.

Item	9-Month-Old Kids		12-Month-Old Kids		*p*-Value
	$\bar{x}$	SD	$\bar{x}$	SD	
Initial weight (kg)	15.58	1.25	16.14	0.97	0.7549
Final weight (kg)	24.06 a	3.76	30.71 b	3.79	0.0110
Daily gains (g)	142.76	48.98	103.80	35.51	0.1227
Cold carcass weight (kg)	9.09 A	1.36	13.22 B	2.18	0.0021
Dressing percentage (%)	37.89 a	2.55	41.27 b	2.23	0.0151
Weight of valuable cuts (kg)					
Entrecote ^1^	0.41	0.07	0.42	0.09	0.5959
Saddle ^2^	0.27 A	0.07	0.48 B	0.11	0.0017
Leg ^3^	1.01 a	0.15	1.54 b	0.27	0.0016
Leg tissue composition (kg)					
Meat	0.74	0.10	0.88	0.28	0.1835
Adipose	0.06	0.02	0.07	0.02	0.2758
Bones	0.17 A	0.05	0.37 B	0.07	0.0001

Explanations: $\bar{x}$—mean; SD—standard deviation; A, B—*p* ≤ 0.01; a, b—*p* ≤ 0.05; means in rows and denoted using different letters differ statistically significantly. ^1^ The entrecote (cutlet) is the middle part of the thoracic part of the carcass. ^2^ The saddle is the lumbar part of the half-carcass. ^3^ The leg is the upper part of the hind limb together with the buttock part of the half-carcass.

**Table 2:** Chemical composition of kid’s meat.

Item	9-Month-Old Kids		12-Month-Old Kids		*p*-Value
	$\bar{x}$	SD	$\bar{x}$	SD	
Moisture (%)	76.31	0.65	76.77	0.33	0.1566
Fat (%)	2.27 a	0.25	1.95 b	0.31	0.0304
Protein (%)	20.71	0.71	20.97	0.22	0.3564
Ash (%)	1.17	0.03	1.18	0.05	0.9457
Vitamin A (µg/g)	0.32	0.05	0.13	0.04	0.1763
Vitamin E (µg/g)	1.32	0.32	1.17	0.32	0.4686

Explanations: $\bar{x}$—mean; SD—standard deviation; a, b—*p* ≤ 0.05; means in rows and denoted using different letters differ statistically significantly.

**Table 3:** Physicochemical properties of kid’s meat.

Item	9-Month-Old Kids		12-Month-Old Kids		
	$\bar{x}$	SD	$\bar{x}$	SD	*p*-Value
pH45	6.25 A	0.25	6.54 B	0.21	0.0192
L* (lightness)	44.38 A	4.32	42.22 B	2.53	0. 0835
a* (redness)	19.28	2.18	18.95	2.53	0. 6998
b* (yellowness)	3.83 A	1.27	2.98 B	1.54	0. 0904
Drip loss (%)	0.10	0.08	0.12	0.07	0.8283
Cooking loss (%)	33.58	7.53	34.11	6.48	0.8817
Shear force (N)	73.56	35.11	66.71	27.23	0.5944

Explanations: $\bar{x}$—mean; SD—standard deviation; A, B—*p* ≤ 0.01; means in rows and denoted using different letters differ statistically significantly.

**Table 4:** Fatty acid composition in intramuscular fat from kid’s meat (g/100 g of lipid).

Item	9-Month-Old Kids		12-Month-Old Kids		
	$\bar{x}$	SD	$\bar{x}$	SD	*p*-Value
C10	0.145	0.035	0.117	0.021	0.0926
C12	0.087	0.047	0.073	0.013	0.4041
C14	1.581	0.317	1.164	0.191	0.1215
C16	18.521 a	1.065	17.103 b	1.021	0.0305
C16-1	1.323	0.221	1.206	0.343	0.2221
C18	16.156	0.525	16.271	2.017	0.4872
C18-1	38.730 a	2.237	34.163 b	3.887	0.0199
C18-2	9.233 A	1.302	12.871 B	1.486	0.0009
Gama 18-3	0.096	0.013	0.098	0.017	0.8549
C20	0.065 A	0.012	0.087 B	0.017	0.0040
C18-3	1.353	0.218	1.249	0.129	0.0784
CLA c9-t11	0.581 A	0.078	0.163 B	0.053	0.0000
CLA t10-c12	-	-	0.016	0.012	0.0084
CLA c9-c11	0.003 A	0.001	0.021 A	0.009	0.0066
CLA t9-t11	-	-	0.031	0.011	0.0001
C22	0.016 A	0.009	0.061 B	0.015	0.0014
C20-4	10.652 a	1.803	14.132 b	2.658	0.0260
C22-1	0.054 A	0.024	0.012 B	0.004	0.0016
EPA	1.033	0.158	0.867	0.163	0.1246
DHA	0.276	0.051	0.289	0.103	0.7681
SFA	36.084	1.451	34.879	2.101	0.2788
UFA	63.933	1.452	65.121	2.101	0.2788
MUFA	40.715 a	2.244	35.380 b	4.165	0.0202
PUFA	23.221 A	3.088	29.740 B	3.941	0.0097
PUFA-6	19.869 A	3.041	27.101 B	3.742	0.0043
PUFA-3	2.752	0.283	2.406	0.355	0.0979
DFA	79.555 A	1.405	95.671 B	9.162	0.0017
OFA	20.316 A	1.417	18.608 B	1.161	0.0358
UFA/SFA	1.778	0.115	1.875	0.173	0.2782
DFA/OFA	3.815 A	0.342	5.169 B	0.685	0.0025
MUFA/SFA	1.131	0.062	1.019	0.148	0.1239
PUFA/SFA	0.648 A	0.112	0.856 B	0.133	0.0153
PUFA 6/3	7.364 A	1.152	11.263 B	1.311	0.0002

Explanations: $\bar{x}$—mean; SD—standard deviation; A, B—*p* ≤ 0.01; a, b—*p* ≤ 0.05; means in rows and denoted using different letters differ statistically significantly. CLA—conjugated linoleic acid, EPA—eicosapentaenoic fatty acids, DHA—docosahexaenoic fatty acids, SFA—saturated fatty acids, UFA—unsaturated fatty acids, MUFA—monounsaturated fatty acids, PUFA—polyunsaturated fatty acids, DFA—hypocholesterolemic fatty acids, OFA—hypercholesterolemic fatty acids.

**Table 5:** Sensory evaluation (points) of samples of kid’s meat after roasting.

Item	9-Month-Old Kids		12-Month-Old Kids		
	$\bar{x}$	SD	$\bar{x}$	SD	*p*-Value
Aroma	4.25 A	0.55	4.53 B	0.45	0.0013
Juiciness	4.09 A	0.46	4.42 B	0.46	0.0004
Tenderness	4.18 A	0.52	4.51 B	0.51	0.0006
Taste	4.27 A	0.45	4.56 B	0.44	0.0034

Explanations: $\bar{x}$—mean; SD—standard deviation. A, B—*p* ≤ 0.01; means in rows and denoted using different letters differ statistically significantly.

## Data Availability

The data presented in this study are available on request from the corresponding author.
